# Pleiotropic Functions of Nitric Oxide Produced by Ascorbate for the Prevention and Mitigation of COVID-19: A Revaluation of Pauling’s Vitamin C Therapy

**DOI:** 10.3390/microorganisms11020397

**Published:** 2023-02-03

**Authors:** Hideo Yamasaki, Hideyuki Imai, Atsuko Tanaka, Joji M. Otaki

**Affiliations:** Faculty of Science, University of the Ryukyus, Nishihara 903-0213, Okinawa, Japan

**Keywords:** antiviral activity, COVID-19, l-ascorbic acid: Linus Pauling, nitric oxide, nitrite, SARS-CoV-2, vitamin C

## Abstract

Linus Pauling, who was awarded the Nobel Prize in Chemistry, suggested that a high dose of vitamin C (l-ascorbic acid) might work as a prevention or treatment for the common cold. Vitamin C therapy was tested in clinical trials, but clear evidence was not found at that time. Although Pauling’s proposal has been strongly criticized for a long time, vitamin C therapy has continued to be tested as a treatment for a variety of diseases, including coronavirus infectious disease 2019 (COVID-19). The pathogen of COVID-19, SARS-CoV-2, belongs to the β-coronavirus lineage, which includes human coronavirus, severe acute respiratory syndrome (SARS), and Middle East respiratory syndrome (MERS). This review intends to shed new light on vitamin C antiviral activity that may prevent SARS-CoV-2 infection through the chemical production of nitric oxide (NO). NO is a gaseous free radical that is largely produced by the enzyme NO synthase (NOS) in cells. NO produced by upper epidermal cells contributes to the inactivation of viruses and bacteria contained in air or aerosols. In addition to enzymatic production, NO can be generated by the chemical reduction of inorganic nitrite (NO_2_^−^), an alternative mechanism for NO production in living organisms. Dietary vitamin C, largely contained in fruits and vegetables, can reduce the nitrite in saliva to produce NO in the oral cavity when chewing foods. In the stomach, salivary nitrite can also be reduced to NO by vitamin C secreted from the epidermal cells of the stomach. The strong acidic pH of gastric juice facilitates the chemical reduction of salivary nitrite to produce NO. Vitamin C contributes in multiple ways to the host innate immune system as a first-line defense mechanism against pathogens. Highlighting chemical NO production by vitamin C, we suggest that controversies on the therapeutic effects of vitamin C in previous clinical trials may partly be due to less appreciation of the pleiotropic functions of vitamin C as a universal bioreductant.

## 1. Introduction

COVID-19 is the coronavirus infectious disease that has brought about a global pandemic since December 2019. As of December 2022, more than six million people have died worldwide due to COVID-19. This pandemic is caused by severe acute respiratory syndrome coronavirus 2 (SARS-CoV-2), which binds to a cell surface receptor of the host, called angiotensin converting enzyme 2 (ACE2) [1]. In a critical presentation of COVID-19, lung injury often progresses rapidly with acute respiratory distress syndrome (ARDS), followed by multiple organ failure due to a “cytokine storm”, a condition of uncontrolled systemic hyperinflammation induced by excessive cytokines [2]. Acute hypoxemic respiratory failure (AHRF) is the most common form of organ failure [3], contributing to over 90% of COVID-19-related deaths in intensive care unit (ICU) patients [4]. The incidence of COVID-19-related AHRF reached 15% of hospitalized patients [5]. Such severe patients with COVID-19 require invasive mechanical ventilation or ECMO (extracorporeal membrane oxygenation) [6].

The pandemic has allowed for continuous mutations of SARS-CoV-2, especially in the spike (S) gene [7], generating further diversity with an evolutionary rate between 0.0004 and 0.002 mutations per nucleotide per year [8]. The World Health Organization (WHO) designated the variants of concern (VOCs) using the Greek alphabet from Alpha, and now it is Omicron [9]. Until recently, a variety of drugs have been applied to cure moderate to severe COVID-19 patients [10,11,12]. Many types of vaccines have been administered to millions of people to suppress the outbreak [13,14]. To date, however, there are few drugs or vaccines available that are specific to new variants.

The antiviral drug remdesivir [10], along with dexamethasone (a systemic steroid) [15], is commonly used in patients with severe forms of COVID-19. Unfortunately, antiviral drugs that are currently available to clinicians have little effect on mortality, the length of in-hospital stay, and the need for mechanical ventilation [5]. COVID-19 and the common cold both present a syndrome of disease states. It may be difficult to rely on a single drug or chemical to cure the disease. High-dose intravenous vitamin C (HDIVC) treatment has been reported to be effective in decreasing days of hospitalization, ICU stay and mortality [16,17,18]. Vitamin C therapy has been known for several decades to be a safe adjunctive treatment that has been examined for a wide variety of diseases, including severe acute respiratory syndrome (SARS) caused by SARS-CoV [19]. Despite its long research background, there are controversies regarding the therapeutic effects of HDIVC treatment [20]. Here, we review ascorbate-dependent nitric oxide (NO) production, which may contribute to mitigating COVID-19 symptoms, as well as preventing SARS-CoV-2 infection. Revisiting Pauling’s vitamin C therapy proposed in the 1970s, the potential therapeutic roles of NO, nitrate, nitrite, vitamin C, and vitamin P are discussed in relation to their pleiotropic functions that have sometimes led to states of confusion.

## 2. NO Therapy for COVID-19 Patients

Inhaled nitric oxide gas (iNO) is considered a safe treatment for hospitalized patients [5,21]. NO is a free radical that plays an important pathophysiological role in the human cardiovascular and immune systems [22,23,24]. The use of iNO was first approved in 1999 by the U.S. Food and Drug Administration (FDA) as a treatment for neonatal pulmonary hypertension [25,26]. In 2003, iNO was tested in severe acute respiratory syndrome (SARS) patients. Clinical trials showed beneficial effects such as decreased pulmonary hypertension, improved arterial oxygenation, and a reduced spread and density of lung infiltrates [27]. Therefore, it was a logical rationale to investigate the therapeutic effects of iNO for COVID-19 as a preventive measure, a treatment against COVID-19, and a rescue treatment to improve arterial oxygenation against ARDS [22,28].

Pregnant patients are at risk of developing AHRF due to pregnancy. A recent cohort study reported that iNO was associated with a reduced need for oxygen supplementation and a shorter hospital stay for pregnant patients with COVID-19 pneumonia in the absence of adverse events in mothers or infants [29], supporting the safety of iNO therapy. Although there have yet to be active debates on the effect of iNO on the clinical outcomes of COVID-19 patients [30,31], a number of studies have confirmed that iNO therapy is associated with improved oxygenation parameters in COVID-19 patients [5,21,32]. The combination use of iNO with other drugs has also been tested as a rescue therapy in COVID-19 patients with ARDS [32].

In iNO therapy, NO can be delivered directly to the pulmonary vasculature by inhalation with a commercially available NO delivery system [23]. In healthy conditions, a sufficient amount of NO should be synthesized by the endogenous enzyme NO synthase (NOS; EC 1.14.13.39) [33]. The principle of iNO therapy can be attributed to a supplementation of NO gas to the pulmonary vasculature to improve the local NO bioavailability. In addition to the inhalation of NO gas, other clinical options to increase NO levels in COVID-19 patients have been investigated to date: the administration of the NOS substrates l-arginine [34,35] and l-citrulline [36] and the administration of NO chemical donors such as nitroglycerin [37], *S*-nitroso-*N*-acetylpenicillamine (SNAP) [38], sodium nitroprusside (SNP) [39], and 1,2,5-oxadiazole-2-oxide (furoxan) [40]. Viagra (sildenafil citrate) is the first commercially available drug for male erectile dysfunction (ED) [41]. Sildenafil, in principle, inhibits phosphodiesterase type 5 (PDE5) which breaks the second messenger cyclic GMP (cGMP), thereby allowing for the accumulation of cGMP in cells even under conditions of low NO input [41]. In many countries, sildenafil has been approved as a treatment for pulmonary hypertension in neonates [42], and its therapeutic effects have also been tested in COVID-19 patients [43,44,45]. These clinical trials have focused on the potent vasodilation activity of NO, which may reduce the risk of respiratory failure.

## 3. Antiviral Activity of NO

### 3.1. Smokers’ Paradox

Asthma and cigarette smoking, as well as obesity, diabetes, and chronic heart disease, are considered high-risk factors for acquiring COVID-19 or poorer outcomes. Early in the pandemic, however, there were few asthma patients with severe cases of COVID-19 [46,47]. A recent meta-analysis study also supported that people with asthma have a lower risk of SARS-CoV-2 infection than those without asthma [48]. The use of inhaled corticosteroids might partly account for a protective effect against SARS-CoV-2 infection, due to decreased ACE2 in asthma patients [49]. It is important to note that NO emission is generally high due to eosinophilic airway inflammation in asthmatic patients. In fact, the fraction exhaled NO (FE_NO_) has been adopted as a non-invasive indicator of the type 2 airway inflammation of asthma [50]. Although non-allergic asthma (non-type 2) seems to have a greater risk, there were fewer asthma patients with COVID-19 in many countries [47], implying that high NO emission from the airway may protect against SARS-CoV-2 infection.

Cigarette smoking has also been listed as a risk factor for contracting COVID-19. In general, cigarette smoking is associated with relatively poor outcomes in respiratory infectious diseases [51]. In 2020, the WHO and the FDA released statements warning that smoking may increase the risk and severity of COVID-19. However, only a low proportion of smokers suffered from SARS-CoV-2 infection [52]. This is referred to as the “smokers’ paradox” [53]. Although smoking cannot be recommended as a protective measure for COVID-19, the underlying mechanism for the smokers’ paradox may give a clue for our consideration of preventing SARS-CoV-2 infection. Farsalinos et al. proposed that nicotine intake could be the reason for the low prevalence of smoking among hospitalized patients [51], whereas Hedenstierna et al. hypothesized that the short burst of concentrated NO (approximately 250 to 1350 ppm per puff) contained in cigarette smoke may prevent SARS-CoV-2 infection [54], an explanation similar to that given for why asthmatic patients are less likely to contract COVID-19.

### 3.2. RNS Biochemistry

**Figure 1:**
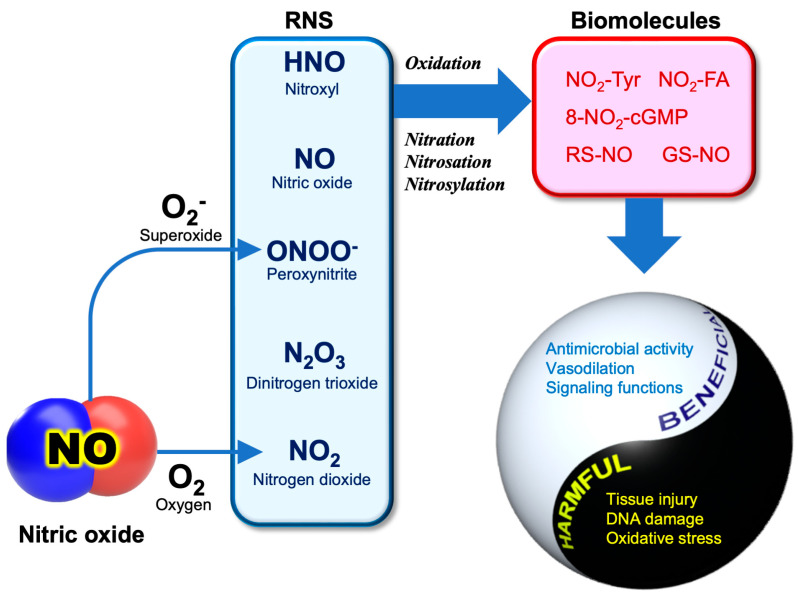
NO and RNS in COVID-19. NO and its derived reactive molecules are frequently referred to as “reactive nitrogen species” (RNS). Peroxynitrite (ONOO^−^) is a reaction product between NO and superoxide (O_2_^−^). RNS potentially mediate the oxidation, nitration, nitrosation and nitrosylation of biomolecules. Those reactions exhibit both beneficial and harmful effects. NO_2_-Tyr, nitro-tyrosine; 8-NO_2_-cGMP, 8-nitroguanosine 3′,5′-cyclic monophosphate; NO_2_-FA, nitro-fatty acids; RS-NO, *S*-nitrosothiol; GS-NO, *S*-nitrosoglutathine.

Apart from its potent actions on cardiovascular systems, NO is involved in innate immunological host defense. The innate immune response is also mediated by reactive oxygen species (ROS), including O_2_^−^, H_2_O_2_, and hypochlorite anion (OCl^−^), which are produced by phagocytic cells such as neutrophils and activated macrophages [55]. Its action is non-specific, and potentially inactivates a broad range of pathogens, including parasites, fungi, bacteria, and viruses [56]. As ROS is the term for a group of reactive molecules derived from O_2_, reactive molecules originating from NO are referred to as “reactive nitrogen species (RNS)” [57]. Figure 1 illustrates the pathophysiological conditions associated with the major RNS.

Like ROS, RNS are highly reactive oxidants that are associated with oxidative stress in living organisms. Compared with ROS, the chemistry of RNS-related reactions is much more complicated, especially under in vivo conditions, and most of them are not fully understood. Basically, the reactions of NO are involved in the oxidation, nitration (the addition of NO_2_), nitrosation (the addition of NO^+^), and nitrosylation (the addition of NO) of biomolecules [58]. An uncontrolled situation of these reactions may cause “nitrative” or “nitrosative stress”, leading to cellular damage or cell death.

Most classes of biomolecules, including proteins, nucleic acids, and lipids, can be nitrated, generating products such as nitro-tyrosine (NO_2_-Tyr) [59], 8-nitroguanosine 3′,5′-cyclclic monophosphate (8-nitro-cGMP) [60], nitro-fatty acids (NO_2_-FA) [61], and nitro-phenolics [62]. NO^+^ could directly react with the thiols (RSH) of cysteine residues or the reduced form of glutathione (GSH) to produce *S*-nitrosothiols (RS-NO, GS-NO). Regarding RS-NO chemical generation in biological systems, several possible mechanisms have been proposed, but currently none of them have reached a consensus [63]. The antipathogenic activity of NO relies on these unique RNS reactions (nitration, nitrosation, and nitrosylation) that are capable of inactivating or killing pathogens through the modification of biomolecules, including enzyme proteins. It is important to remember that these reactions are non-specific, thereby also causing cellular damage to the host cells during inflammation [64]. It appears that the use of RNS as a countermeasure against pathogens is a risky business for hosts.

Under oxidative stress conditions where ROS are overproduced, such as during inflammation, peroxynitrite (ONOO^−^) can be produced as the reaction product between NO and O_2_^−^, an important interplay between ROS and RNS [65].

The rate constant for the reaction between NO and O_2_^−^ is near diffusion controlled (Equation (1)), which is faster than the superoxide dismutase (SOD) reaction that removes O_2_^−^. The product ONOO^−^ is stable at pH 12 in the absence of target molecules. At physiological pH, ONOO^−^ is in rapid equilibrium with its conjugated acid peroxynitrous acid (ONOOH, p*K*_a_ 6.8) (Equation (2)), which is a short-lived molecule that spontaneously decays to nitrate (Equation (3)) [66]. Due to its high reactivity, ONOO^-^ is considered the major cytotoxic agent in RNS.

NO + O_2_^−^ → ONOO^−^ (1)

ONOO^−^ + H^+^ → ONOOH (2)

ONOOH → NO_3_^−^ + H^+^ (3)

ONOO^−^ and ONOOH are strong oxidants capable of oxidizing various molecules, such as thiols, sulfides, ascorbate, and phenols [67]. In addition to the oxidation of molecules, ONOO^−^ can chemically nitrate aromatics, with a reaction being facilitated in the presence of bicarbonate anion (HCO_3_^−^) [68]. The dysfunction of proteins or enzymes may occur due to the formation of nitro-tyrosine residues (NO_2_-Tyr). ONOO^-^ is also involved in DNA fragmentation [67] and RNA viral mutation [69] through deamination of the bases.

### 3.3. Anti-SARS-CoV-2 Activity of NO

The antiviral activity of NO has been reported for many types of viruses, most typically, DNA viruses such as murine poxvirus, herpesviruses, and some RNA viruses [55]. The direct action of NO as an antiviral agent involves the inhibition of viral replication and viral entry into the host [70,71]. In 1999, Saura et al. demonstrated that the in vitro replication of the RNA virus coxsackievirus is suppressed by NO-dependent *S*-nitrosylation that causes the inactivation of viral cysteine protease, an enzyme necessary for replication [72]. The *S*-nitrosylation of the cysteine-containing enzymes of viruses is thought to be a general mechanism for the antiviral activity of NO [73].

SARS-CoV-2 is a positive-sense RNA virus belonging to the family Coronaviridae, which includes severe acute respiratory syndrome coronavirus (SARS-CoV), the pathogen that caused the SARS outbreak. In 2005, Akerstrom et al. reported that the NO chemical donor SNAP inhibits the in vitro replication cycle and the protein and RNA synthesis of SARS-CoV [74]. This inhibitory effect was not observed with SNP (sodium nitroprusside), another chemical NO donor [38]. Likewise, NO released from SNAP was reported to inhibit the replication of SARS-CoV-2 in Vero E6 cells through the inhibition of the SARS-CoV-2 3CL cysteine protease [75].

Macrophages are multifunctional innate immune cells that play an essential role in the clearance of pathogens and control inflammatory responses. Recent studies have suggested that *S*-palmitoylation is a key reaction for control macrophages in the processes of endocytosis [76]. Interestingly, NO was reported to suppress the palmitoylation of the spike (S) proteins that is needed for their binding to ACE2 [77]. The spike (S) proteins of coronaviruses are receptor-binding proteins that are synthesized in the endoplasmic reticulum (ER), followed by complex post-translational modification in the host Golgi apparatus [78,79,80]. *S*-Palmitoylation is one of the post-translational modifications in the Golgi apparatus where palmitoyl acyltransferase (PAT) adds the saturated fatty acid palmitate (C16:0) to the cysteine thiol group (-SH) of proteins [78,80]. Protein modification causes an increase in the hydrophobicity of the proteins, which is essential for cell-cell fusion activity [79,81]. Endothelial NO synthase (eNOS), an isoform of the host’s NO-producing enzyme, can be modified by palmitoylation, and its activity is decreased by the modification [82]. *S*-nitrosylation of the SARS-CoV spike (S) protein with NO may reduce cell-cell fusion activity through decreased amounts of spike (S) protein palmitoylation [77]. It is presumable that the disturbance of the cysteine palmitoylation of the spike (S) proteins is also involved in the mechanism for antiviral activity of NO against the coronavirus [83].

## 4. Diversity of NO Generating Mechanisms

### 4.1. Oxidative and Reductive Mechanisms for NO Generation

It is evident that NO exhibits substantial antiviral activity against many types of viruses, including SARS-CoV-2. In mammals, NOS plays a central role in endogenous NO supply. Notably, the NOS reaction is not the only endogenous source of NO [84,85]. There are multiple mechanisms for biological NO production other than NOS. Such distinct mechanisms have been called the “nitrate-nitrite-NO pathway” [86,87,88], “NOS-independent mechanism” [89], “nitrite-dependent NO production pathway” [65,90], “nitrite pathway” [91], or the “reductive pathway” [92].

**Figure 2:**
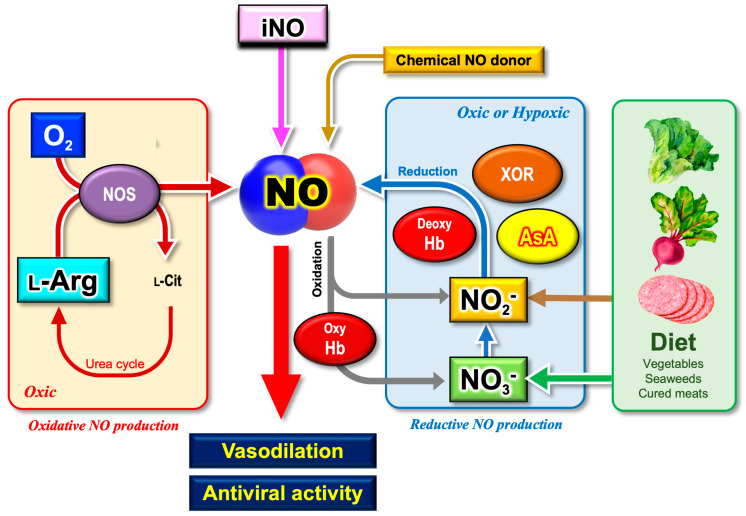
Diversity of NO generation mechanisms. NO displays multiple physiological functions in humans, such as vasodilation effects. It has also been suggested that NO inhibits the replication of viruses, including SARS-CoV, thereby preventing viral infection. There are two distinct mechanisms for NO synthesis, namely, NOS-dependent and NOS-independent NO generating mechanisms. Regardless of the pathways, inorganic nitrate (NO_3_^−^) and/or nitrite (NO_2_^−^) are produced as the oxidation product of NO. In humans, NO_3_^−^ is supplied by daily diets, including green vegetables or seaweeds, which may help to support NO bioavailability. NO, nitric oxide; iNO, inhaled nitric oxide; NOS, nitric oxide synthase; oxy-Hb, oxy-hemoglobin; deoxy-Hb, deoxy-hemoglobin; XOR, xanthine oxidoreductase; NO_2_^−^, inorganic nitrite; NO_3_^−^, inorganic nitrate; AsA, l-ascorbic acid (vitamin C).

It has been demonstrated by many researchers that inorganic nitrite (NO_2_^−^) is reduced to NO by redox enzymes or reductants. This alternative mechanism for NO production (hereafter the reductive mechanism) appears to be less appreciated than the NOS-dependent NO production mechanism (hereafter the oxidative mechanism) [92]. Figure 2 summarizes our current knowledge on the multiple sources of NO, relevant to the pathophysiology of COVID-19.

### 4.2. Nitrite as a Degradation Product of NO

NOS utilizes the amino acid l-arginine (l-Arg) and molecular oxygen (O_2_) as substrates and requires nicotinamide-adenine-dinucleotide phosphate (NADPH), flavin adenine dinucleotide (FAD), flavin mononucleotide (FMN), and (6R-)5,6,7,8-tetrahydro-l-biopterin (BH_4_) as cofactors [33] (Equation (4)):

l-arginine + NADPH + H^+^ + O_2_ → l-citrulline +1/2 NADP^+^ + NO + H_2_O (4)

There are three isoforms of NOS in mammals: neural NOS (nNOS, NOS1), inducible NOS (iNOS, NOS2), and endothelial NOS (eNOS, NOS3) [33]. nNOS is expressed in specific neurons of the central nervous system (CNS), where NO acts as an atypical neurotransmitter associated with learning and memory formation [33]. NO formed in the CNS by nNOS is involved in the central regulation of blood pressure. The inhibition of nNOS activity in the medulla and hypothalamus results in systemic hypertension [66]. eNOS plays a key role in vascular functions, which mediates basal vasodilation [93]. NO generated by iNOS exhibits beneficial antiviral activity, whereas aberrant iNOS induction is also associated with many types of diseases, including asthma, arthritis, multiple sclerosis, colitis, neurodegenerative diseases, tumor development, and septic shock [94].

Although the autoxidation of NO is a kinetically slow reaction [92], nitrite (NO_2_^−^) can be produced as an oxidation product of NO in the presence of O_2_ (Equation (5)):

4NO + O_2_ +2H_2_O → 4NO_2_^−^ + 4H^+^ (5)

The Griess method has been widely used to quantify NOS activities [95]. The method was originally designed to detect nitrite (NO_2_^−^), as well as nitrate (NO_3_^−^), in a solution [96], and it was later applied to estimating NO production in biological samples [97]. In addition to the autoxidation of NO, nitrate (NO_3_^−^) can be produced by NO-dioxygenation, which is mediated by blood oxyhemoglobin (Oxy-Hb) [92] (Equation (6)):

NO + Hb (Fe^II^-O_2_) → NO_3_^−^ + Hb (Fe^III^) (6)

Here, it should be emphasized that inorganic nitrite (NO_2_^−^) and nitrate (NO_3_^−^) are produced as the degradation products of NO, which is why the Griess method has been widely used as an index parameter of NOS activity.

### 4.3. Nitrite as a Precursor of NO

Antimicrobial technology is important for food industries in the prevention of food poisoning. In many countries, nitrite is ordinarily contained as a food additive in cured meats such as in sausage, ham, or bacon. When meats are processed with nitrite salt, the meat can be stored at room temperature for a long time without degrading. The mechanism for the antibacterial activity of nitrite was uncovered by the discovery of NO production from nitrite. It is not nitrite but NO that functions as an antibacterial agent that prevents the food poisoning of cured meats.

The inorganic nitrite ion (NO_2_^−^) exists in equilibrium with its conjugate acid, nitrous acid (HNO_2_, p*K_a_* 3.1–3.5) (Equations (7)–(8)). HNO_2_ spontaneously produces dinitrogen trioxide (N_2_O_3_) as a result of the disproportionation reaction (Equation (9)). NO can be generated by the chemical decomposition of N_2_O_3_ [98] (Equation (10):

NO_2_^−^ + H^+^ ←→ HNO_2_ (7)

3HNO_2_ → 2NO + HNO_3_ + H_2_O (8)

2HNO_2_ → N_2_O_3_ + H_2_O (9)

N_2_O_3_ → NO + NO_2_ (10)

Since spontaneous NO production from nitrite under acidic conditions is relatively slow [65], it may have minimal physiological relevance, such as in gastric juice [99].

### 4.4. Saliva as the Major Source of Nitrite

The presence of endogenous nitrite formation in the human body was first noted by Mitchell et al. (1916) more than a century ago [100], but the endogenous source of this nitrite had remained unknown until the 1980s [90]. The discovery of NOS reactions has provided an answer to Mitchell’s observation (Equation 5). In healthy conditions, the consumption of a high-nitrate meal results in a huge increase in the nitrate concentration in plasma, as well as in urine [101]. The exogenously supplied nitrate can be converted to nitrite by the nitrate reductase activity of symbiotic bacteria (Equation (11)):

NO_3_^−^ + 2e^−^ + 2H^+^ → NO_2_^−^ + H_2_O (11)

In humans, nitrate (NO_3_^−^) is highly concentrated by the salivary glands and secreted into saliva [102]. The nitrate concentration in saliva was reported to be a few hundred µmoles/L in basal conditions, but their concentrations increase to a few mmoles/L after the consumption of nitrate [101]. In 2012, Qin et al. demonstrated that sialin (*SLC17A5*) expressed in the plasma membrane of salivary gland acinar cells functions as a nitrate transporter [103]. Sialin (*SLC17A5*) was originally identified as the cause of sialic acid (SA) storage disease or Salla disease [104,105], and the protein has been known to mediate SA/H^+^ cotransport [103]. They found that sialin (*SLC17A5)* is also capable of transporting NO_3_^−^ and H^+^ via an electrogenic cotransport mechanism [103].

The oral ingestion of potassium nitrate (KNO_3_) solution quickly increases the salivary nitrite (NO_2_^−^) concentration [99], which cannot be observed after using a mouthwash to sterilize oral bacteria [106]. The oral cavity has the second largest and diverse microbiota, harboring over 700 species of bacteria [107]. Many commensal bacteria are capable of reducing nitrate (NO_3_^−^) to nitrite (NO_2_^−^) by their nitrate reductase (NR) activity [106]. Approximately 5% to 7% of dietary nitrate is converted to nitrite in the oral cavity by facultative anaerobic bacteria on the posterior part of the tongue [108]. Through spontaneous chemical reactions (Equations 7–10), swallowed salivary nitrite is then converted to NO under the strong acidic conditions of gastric juice in the stomach [109]. Responding to salivary nitrite concentrations, a huge amount of NO can be generated in the stomach by the ascorbate-dependent reductive mechanism described below. This series of reactions has been considered to inactivate swallowed pathogens [98]. Nitrite and acidic conditions are the key factors in antimicrobial activity.

## 5. Ascorbate-Dependent NO Production

### 5.1. Antibacterial Activity of Vitamin C

Urinary acidification has long been used as an aid for the treatment of urinary tract infections (UTI). In 1959, McDonald and Murphy suggested the use of ascorbate as an acidifying agent in the treatment of UTIs. Since UTIs are one of the most frequent complications during pregnancy (between 8 and 23%), even today, daily intake of ascorbate (vitamin C) is recommended, particularly for pregnant women [110]. However, the mechanism for the antibacterial action of vitamin C has long been unclear.

It has long been known in chemistry that NO can be generated by the reduction of nitrite in the presence of iodide or ferrocyanide in an acidified solution. Historically, this reaction was used to quantify nitrate/nitrite in urine [100]. This chemical production of NO via the reductive mechanism is almost stoichiometric, thereby being widely used as a convenient method to calibrate NO concentrations in a solution, even today [90]. Ascorbate (vitamin C, l-ascorbic acid, AsA), a potent natural antioxidant contained in fruits and vegetables [111], has also been reported to induce the chemical NO production from nitrite [112]. The one-electron reduction of nitrite produces NO and the short-lived monodehydroascorbate (MDA) radical (Equation (12)):

2HNO_2_ + 2AsA → 2NO + 2MDA + 2H_2_O (12)

Two molecules of MDA spontaneously disproportionate to ascorbate and dehydroascorbate (DHA) (Equation (13)):

2MDA → AsA + DHA(13)

Plants have evolved to reduce MDA radicals with the MDA reductase (MADR) enzyme to regenerate ascorbate [113], but no specific enzyme for MDA reduction has been identified in animal cells. As a result of these reactions, two molecules of NO can be produced by ascorbate (Equation (14)):

2HNO_2_ + AsA → 2NO + DHA + 2H_2_O (14)

The antibacterial function of ascorbate in urine may include the chemical NO generation from the nitrite contained in urine [114].

### 5.2. Ascorbate Secreted from the Stomach

Ascorbate is known to be secreted into the stomach, leading to gastric juice concentrations greater than those in plasma [115]. It is also known that the secretion of ascorbate is impaired in the presence of chronic gastritis [116]. It has long been puzzled what the physiological functions of the secretion of ascorbate into gastric juice might be. Epidemiological investigations have suggested that ascorbate in gastric juice protects us from gastric cancer [116]. It was hypothesized that ascorbate in gastric juice may be required for the chemical reduction of carcinogenic *N*-nitroso compounds, such as the nitroso amines implicated in gastric carcinogenesis, a possible mechanism for the prevention of stomach cancer by ascorbate [117,118].

NO has been detected in the gastric headspace after nitrate ingestion [109]. Its concentration was reported to be between 14.8 and 89.4 ppm after potassium nitrate (KNO_3_) ingestion [98]. Taking the presence of nitrite in saliva fluid into account, one can expect that ascorbate secreted into gastric juice facilitates the efficient chemical reduction of salivary nitrite to generate a ppm level of NO in the stomach [119]. A strong acidity (approx. pH 1 in fasting conditions) of gastric juice is considered the first defense mechanism against the oral transmission of pathogens. However, this strong acidity cannot be retained after the ingestion of foods. Chemical NO production with nitrite and ascorbate may further ensure the inactivation of pathogens through the antimicrobial activity of NO, even after the ingestion of foods.

### 5.3. Plants as the Major Dietary Source of Nitrate and Vitamin C

Vegetables are the major dietary sources of nitrate for humans (Figure 2). Approximately 80% of ingested nitrate can be attributed to vegetables [108]. Absorbed by roots in soils and translocated through the plant vascular system, nitrate can become highly concentrated in plant leaves, i.e., green leafy vegetables [120]. Plants synthesize the amino acids in leaves, where nitrite transported into the chloroplasts is further reduced by light energy to synthesize glutamate (nitrogen assimilation). Nitrate contents vary widely between plant species [121], and even within the same tissue types of the same species due to the differences in nitrate fertilization during cultivation [120]. A hundredfold variation in average nitrate levels was found in cooked greens, ranging from 4850 mg kg^−1^ in English spinach to 48 mg kg^−1^ in iceberg lettuce [122]. Hence, fresh green leafy vegetables are a dietary source of nitrate.

**Figure 3:**
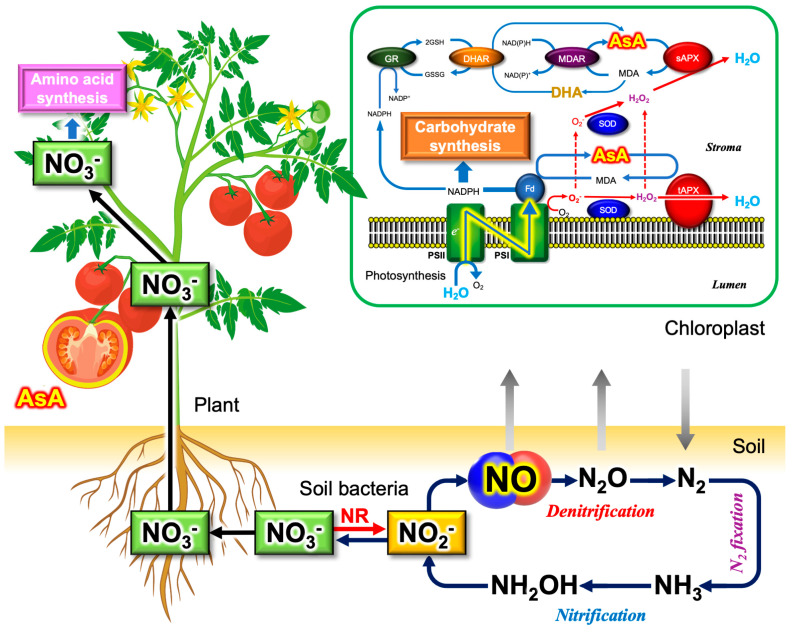
Nitrate and vitamin C in vegetables and fruits. On Earth, plants and soil bacteria sustain the global nitrogen (N) cycle, which is essential for recycling the N contained in biomolecules, including proteins and nucleic acids. NO is involved in this global cycle through the denitrification activities of soil bacteria. Both denitrifying and nitrifying bacteria produce inorganic nitrite (NO_2_^−^) as an intermediate metabolite [124,125,126]. NO_3_^−^ is absorbed by the roots, followed by being delivered to the green leaves where photosynthetic CO_2_ assimilation takes place. NO_3_^−^ is reduced to NO_2_^−^ in the cytosol and further reduced to glutamate using light energy. In addition to nitrogen assimilation, chloroplasts are involved in the synthesis of cysteine using light energy. During the three major assimilation metabolisms, ROS, RNS, and RSS are produced as the byproducts [127], which is a strong reason why green leaves contain potent antioxidant systems [128]. In plant cells, NO is produced by reductive mechanisms [91,129,130]. Ascorbate accumulates in fruits and leaves. In leaves, ascorbate is present at high concentrations in the chloroplast stroma, such as 25 mM [131]. Ascorbate in the chloroplast is essential to detoxify ROS produced during the photosynthetic electron transport. Ascorbate is used to remove H_2_O_2_ by ascorbate peroxidase (APX), and the oxidized ascorbate monodehydroascorbate (MDA) and dehydroascorbate (DHA) are quickly reduced to regenerate ascorbate using light energy. The inset shows a schematic diagram for the water‒water cycle [132]. Ascorbate also accumulates in fruits at high concentrations. In tomato fruits, the ascorbate content in red fruits is higher than that in green fruits [133]. The localization of ascorbate in a tomato fruit is shown in yellow. AsA, l-ascorbic acid (vitamin C); MDA, monodehydroascorbate; DHA, dehydroascorbate; tAPX, thylakoidal ascorbate peroxidase; sAPX, stromal ascorbate peroxidase; MDAR, monohydroascorbate reductase; DHAR, dehydroascorbate reductase; GR, glutathione reductase; SOD, superoxide dismutase; Fd, ferredoxin, PSI, photosystem I complex; PSII, photosystem II complex; NR, nitrate reductase.

Vitamin C can be acquired from green leafy vegetables and fruits. In the 1920s to 1930s, Albert Szent-Györgyi identified and isolated ascorbate from the fruits of Hungarian red pepper [111]. Since humans do not synthesize ascorbate due to the loss of l-gulono-1,4-lactone oxidase (GULO), diets are extremely important to acquire vitamin C. As with nitrate content, ascorbate content also varies to a large extent between species and tissues [123]. Figure 3 illustrates nitrate and vitamin C in plants (vegetables and fruits).

Plants synthesize ascorbate mainly through the Smirnoff–Wheeler pathway, which is dominant in photosynthetic tissues, i.e., green leafy vegetables [111]. However, the reason why plants accumulate high concentrations of ascorbate in leaves had been unknown for a long time. In 1976, Foyer and Halliwell proposed that ascorbate may be involved in a mechanism for the removal of ROS in plants [134]. After the discovery of ascorbate peroxidase (APX) and ascorbate recycling systems, including monodehydroascorbate reductase (MDAR) and dehydroascorbate reductase (DHAR) [135], it became evident that high concentrations of ascorbate are essential to operate the O_2_-evolving photosynthetic electron transport system. Ascorbate is used to detoxify the ROS produced in chloroplasts under illumination [136]. This unique plant ascorbate-dependent ROS scavenging mechanism is referred to as the ascorbate–glutathione cycle [137] or the water‒water cycle [132] (Figure 3). The reason why fruits, which are not photosynthetic organs, accumulate ascorbate remains a subject to be investigated [111].

### 5.4. Vitamin P and NO

In 1936, Szent-Györgyi and coworkers demonstrated that the flavonoid glycosides rich in citrus fruits behave similarly to ascorbate in maintaining capillary permeability. Based on his observation, Szent-Györgyi proposed that plant flavonoids are essential nutrients as vitamin P (permeability) [138], a short-lived vitamin concept [139]. His idea that flavonoids can complement the function of ascorbate has been renewed with the development of the antioxidant hypothesis of phytochemicals.

Flavonoids are representative secondary metabolites of plants, accumulating in the epidermal cells of organs such as flowers, leaves, stems, roots and fruits [139]. Flavonoids can be classified into subgroups: chalcone, flavanone, flavone, isoflavone, flavonol, and anthocyanin. Most of these subgroups exhibit a yellowish coloration, whereas anthocyanins show multiple colorations depending on their structures, the presence of metals, their pH and their conjugation with other molecules [139]. In the 1980s, the anthocyanins contained in red wine were highlighted to account for the paradoxical epidemiological observation called the “French paradox”; French people have a relatively low incidence of coronary heart disease even though they consume a diet relatively rich in saturated fats [140]. Researchers were interested in the anthocyanins or “polyphenols” contained in red wine that may suppress heart disease through their antioxidant activities [140]. It is now widely appreciated that flavonoids or “polyphenols” are beneficial for human health largely due to their non-specific antioxidant functions [141]. In eNOS-dependent NO production (the oxidative mechanism), the flavonoids quercetin and catechin contained in red wine were demonstrated to increase the bioavailability of NO by scavenging O_2_^−^ [142]. In plants, phenolic secondary metabolites, including flavonoid pigments, not only contribute to the plant colorations of flowers and fruits but also suppress oxidative damage of the cells through enzymatic [143] and non-enzymatic [144] mechanisms, complementing the ascorbate antioxidant systems [128].

If plant flavonoids and polyphenolic compounds are functionally equivalent to ascorbate, NO can be chemically generated from nitrite in the presence of plant pigments (Equation (15)):

HNO_2_ + Phe-OH → NO + Phe-O˙ + H_2_O (15)

where Phe-OH and Phe-O˙ are the parent reduced form of phenolic compounds and their phenoxyl radicals, respectively. Gago et al. (2007) explored the involvement of anthocyanins in the reductive mechanism for NO generation in the stomach [145]. Following the consumption of red wine, a higher NO in the air expelled from the stomach (30,215 ppb) was detected than that under fasting conditions (1196 ppb), with a peak at 15 min. This wine effect was not observed in the absence of exogenous nitrate consumption [145]. Rocha et al. (2009) showed in vitro evidence that several classes of flavonoids can reduce nitrite to produce NO at pH 2 [146]. It should be noted that phenoxyl radicals can be efficiently reduced back to their parent compounds by ascorbate [147], implying that complicated redox interplays between vitamin P and vitamin C. Although there have been a limited number of studies available to date [146,148], it appears to be obvious that vitamin P also contributes to the reductive generation of NO in humans.

Plant (vegetable and fruit) coloration is attributed to the spectral properties of flavonoids, mostly anthocyanins. The plant pigment betalains that are included in spinach, chard, and beetroot are exceptional. The term “betalain” comes from the Latin name of the common beet (*Beta vulgaris*) from which betalains were first extracted [139]. Betalains are a class of tyrosine-derived pigments that are distributed in only 13 families of the Caryophyllales order, such as red beet (Amaranthaceae) and cactus (Cactaceae), and in some fungi [149]. To date, anthocyanins and betalains have never been detected jointly in plant tissues [149]. The biological meaning of the mutually exclusive relationship between betalains and anthocyanidins is still unknown [150]. Beetroot juice has recently attracted much attention from researchers as a natural nitrate (NO_3_^−^) supplement [151]. In addition to nitrate supplementation and the antioxidant activity of betalains, the chemical NO-generating activity of betalains with nitrite is of great interest to understand the beneficial effects of beetroot juice [152,153].

## 6. Possible Roles of Nitrate, Nitrite, and Vitamin C in the Prevention of COVID-19

### 6.1. Toxicity of Nitrite and Nitrate

Nitrite has long been recognized as an inorganic toxic compound by food professionals as well as the public, mostly for two reasons: methemoglobinemia (cyanosis or blue baby syndrome) and the formation of nitrosamines. Water supplies, especially near agricultural areas, are often contaminated with nitrate derived from nitrogen fertilizer. Nitrite may be produced in such contaminated water by ambient bacteria via their nitrate reductase activities. In 1945, the pediatric resident Hunter Comly revealed that the use of nitrate-contaminated well water is the direct cause of blue baby syndrome, a condition characterized by cyanosis or blueness of the skin in babies [154]. Meanwhile, another concern regarding the toxicity of nitrite arose in food industries. Nitrite is an important meat preservative, but carcinogenic nitrosamines might be produced during the cooking of cured meats that contain nitrite. Because of these two reasons, the contents of nitrite and nitrate in drinking water, vegetables and cured meats have been strictly regulated by governments and authorities in many countries.

Contrary to common sense, many recent animal and epidemiological studies have not indicated such cytotoxicity, except for in infants. Rather, nitrite is now considered a potential therapeutic agent for the prevention and reversal of cerebral vasospasm after subarachnoid hemorrhage (SAH), the prevention of reperfusion injury associated with myocardial infarction, the elicitation of therapeutic angiogenesis, the treatment of pulmonary hypertension, and the enhanced preservation of tissues for transplantation [155]. Scientists’ appreciation of nitrite (nitrate) in life science has drastically changed from a toxin to a bioactive molecule. The prompt excretion of nitrite and nitrate into urine further supports the safety of nitrite and nitrate. Hence, it is now conclusive that the oral ingestion of nitrate is quite safe, at least as long as it is taken from vegetables and fruits.

### 6.2. Prevention of SARS-CoV-2 Transmission

**Figure 4:**
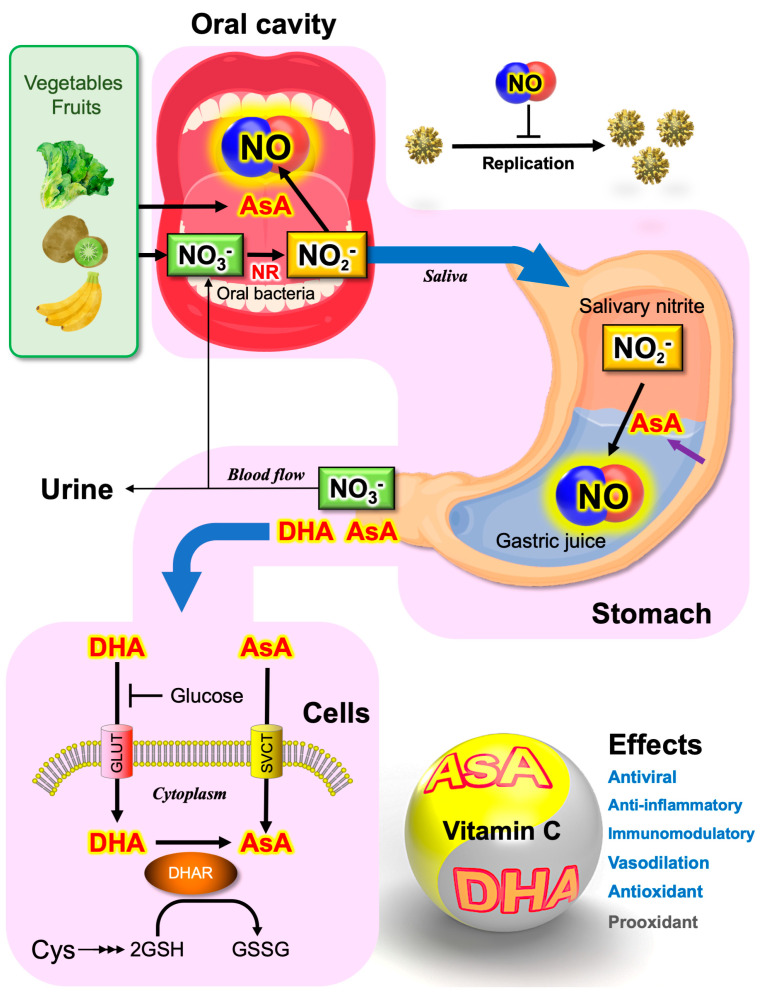
Possible functions of orally-taken ascorbate and nitrate in the prevention and mitigation of COVID-19. Plants (vegetables and fruits) contain abundant nitrate (NO_3_^−^) and vitamin C (l-ascorbate) in their tissues. When chewing those diets with saliva, NO can be generated by the reductive mechanism. Organic acids contained in fruits and vegetables facilitate the chemical production of NO in the oral cavity. The oral cavity harbors a huge diversity of symbiotic bacteria that can reduce the nitrate secreted from saliva glands to nitrite (NO_2_^−^). If powdery vitamin C is taken orally, a high concentration of NO could be generated in the oral cavity by the reductive mechanism. When saliva, including nitrite, is mixed with gastric juice, similarly, chemical NO generation occurs. The strong acidity of the gastric juice facilitates chemical NO generation. NO_3_^−^ is absorbed in the small intestine and is concentrated in the saliva glands. The vitamin C in plasma is incorporated into cells by sodium-dependent Vit C transporters (SVCTs) [20]. In parallel, dehydroascorbic acid (DHA), an oxidized form of vitamin C, is taken up through glucose transporters (GLUTs) [20]. Since glucose competes with DHA on transporters [163], vitamin C availability in cells may be limited in high sugar diets and high blood sugar conditions, a potential reason for the pathological severity of COVID-19 in diabetes patients [164]. Ascorbate and DHA show direct and indirect pharmaceutical effects that are sometimes opposed. AsA, l-ascorbic acid or vitamin C; DHAR, dehydroascorbic acid reductase; GSH, reduced form of glutathione; GSSG, oxidized form of glutathione; Cys, l-cysteine.

Since 2019, most studies have focused on SARS-CoV-2 infection in the respiratory tract due to the close association of respiratory failure with COVID-19-related death, as mentioned in the introduction. However, ACE2 is not only expressed in the lungs but also in multiple organs implicated in COVID-19 complications. A number of studies have reported gastrointestinal symptoms in patients with COVID-19 [156]. The small intestine is one of the organs where high ACE2 expression occurs [157]. A meta-analysis of 4243 patients with COVID-19 from six countries reported that 17.6% of COVID-19 patients had gastrointestinal symptoms, and 48.1% of fecal samples from COVID-19 patients tested positive for viral RNA [158]. It is important to note that the prolonged shedding of viral RNA in stool rather than in respiratory samples was observed in 70.3% of the patients, which could be up to 33 days from illness onset [158]. During the SARS outbreak in 2003, a vertical spread of the outbreak in buildings was reported in the Amoy Gardens, Hong Kong. Researchers have speculated that the sewage system of the Amoy Gardens may have served as the major source of infection from patients excreting SARS-CoV [159,160]. Although there are ongoing active debates on fecal-oral transmission in COVID-19 [156,161], potential gastrointestinal infection should be carefully considered in preventing the outbreak [156,162]. To limit the oral transmission of viruses, as well as aerosol or airborne transmission, we suggest a reductive NO generation strategy for preventing and mitigating COVID-19, as shown in Figure 4.

Gastric juice acts as the first line of defense to avoid gastrointestinal infection by pathogens [165]. Its pH is near 1 (equivalent to 0.16 M HCl [166]). However, this strongly acidic pH can be measured only under fasting conditions. The pH may go up to a near neutral pH after the ingestion of foods. The strong acidity of gastric juice is sustained by the delivery of H^+^ into the lumen of the gastric gland, along with secreted Cl^−^ [166]. H^+^ extrusion is mediated by H^+^, K^+^-ATPase, an important drug target for gastric hyperacidity [167,168]. In 2017, Zhou et al. compared the gastrointestinal fluid tolerance of viruses between MERS-CoV, human coronavirus hCoV-229E, and EV71, a prototype of human enterovirus [169]. They suggested that MERS-CoV cannot be inactivated at a higher pH and that the intestinal tract may serve as an alternative infection route for MERS-CoV, which can be facilitated by the ingestion of foods or inhibition of the H^+^-pump [169].

In 2010, Kopic et al. reported that 2% ethanol significantly suppressed acid secretion through the inhibition of the AMP-activated protein kinase (AMPK), implying that a low dose of ethanol may elevate the pH of gastric juice [168]. There has been a great deal of controversy as to the effects of ethanol on gastric acid secretion, from being a mild stimulant to being a potent inhibitor [166]. Although the impact of ethanol intake on the oral transmission of pathogens has not yet been clarified [170,171], dinner with alcoholic beverages can be considered a risk factor in the oral transmission of pathogens. It is a logical speculation that the combination of ascorbate and nitrite can inactivate pathogens through reductive NO chemical generation, and is presumably effective even after the ingestion of foods (increased pH) and alcoholic beverages (inhibition of the H^+^-pump). 

## 7. Vitamin C Therapy in COVID-19

### 7.1. Free Radical Storm

“It’s not stress that kills us; it is our reaction to it” (Hans Selye, 1956) [172].

The Nobel laureate Hans Selye was a physiologist who established his theory on stress [173]. The stress theory had long been a philosophical concept that was rather difficult to explain on a molecular basis. Infectious diseases, including COVID-19, can be categorized as “biotic stress” due to the biological pathogen-host interactions. According to stress theory, the immune response can be interpreted as a stress response against biological stressors. Indeed, SARS-CoV-2 itself does not kill us, but our immune response “cytokine storm” or hyperinflammation accompanying the overproduction of NO and ROS kills us through organ failure.

NO and ROS are so called “double-edged swords”. In small amounts, they function as signaling molecules in cells, but continuous exposure at high concentrations sometimes damages the host cells themselves. In viral infection, proinflammatory cytokines, such as interferon-γ (INF- γ), upregulate iNOS, which potentially overproduces NO in the pathogenesis of viral infection [55]. NO overproduction may be directly linked to the pathogenesis of viral pneumonia [55]. In fact, the administration of the NOS inhibitor *N^w^*-monomethyl-l-arginine (l-NMMA) or the O_2_^−^ scavenger superoxide dismutase (SOD) protected and recovered influenza virus-infected mice [174,175]. Lab mice deficient in iNOS showed reduced morbidity, mortality, and diminished cytokine production in the lung tissue following an influenza virus challenge [176]. These early experiments clearly indicated that NO, as well as ROS, contribute not only to the host defense but also to the severity of viral pathogenic diseases. As Wu (2020) pointed out, it is not a “cytokine storm” but a “free radical storm” that may be the direct cause of organ failure or cell death in COVID-19 [177].

### 7.2. High-Dose Intravenous Vitamin C Treatment

Along with anti-inflammatory drugs, the use of potent antioxidants may become a clinical option to calm the “free radical storm” in critical conditions. Vitamin C therapy has been known for several decades as a safe adjunctive treatment that has been examined in a wide variety of diseases, including cancer [178], obesity [179], and SARS [19]. Sepsis is a medical emergency that occurs due to the hyper-immunological response of patients. ARDS is a devastating complication. There has recently been emerging evidence for the use of vitamin C as a treatment for sepsis [20]. Similarly, high-dose intravenous vitamin C (HDIVC) treatment has been reported to be effective in decreasing days of hospitalization, ICU stay and mortality in patients with COVID-19 [16,17,18,36]. Bypassing the limitations of vitamin C uptake through a sodium-dependent vitamin C transporter (SVCT1), HDVIC can achieve 70-fold higher plasma concentrations of vitamin C compared to oral administration [180]. Despite its long research background, however, there are controversies regarding the therapeutic effects of HDIVC treatment [20].

Early in the pandemic, the number of COVID-19 patients was apparently low in Japan in contrast with Europe and the U.S., despite its high population density, large percentage of high-risk individuals over 65 years old, and the absence of restricted social activities [181]. The paradoxical phenomenon was often called the “Japanese paradox” [182]. The Japanese paradox received much attention from researchers, as well as from the public at that time. Many hypotheses have been proposed to account for the Japanese paradox in terms of cultural differences, the preacquisition of immunity, genetic background (ACE2 and HLA), and BCG vaccination [183]. As discussed above, it is likely that nitrate-rich dietary foods are involved in the prevention of SARS-CoV-2 infection through a reductive mechanism (Figure 2). In good agreement with this prospect, the Japanese diet containing abundant nitrate has been reported to improve hypertension and other vascular diseases [183]. Despite the promising therapeutic effects of nitrate on NO generation, a recent randomized clinical trial did not support our expectations [184]. Moreover, Lorente et al. (2022) reported higher blood nitrate and nitrite levels in non-survivor COVID-19 patients than in healthy subjects [185], presumably due to the overproduction of NO that produces nitrate and nitrite as the degradation product (Section 4.2). Similar to vitamin C therapy, there have been contradictory results on the therapeutic effects of nitrate and nitrite. It is likely that ascorbate therapy alone, or nitrate (or nitrite) therapy alone, may be insufficient to drive the reductive mechanism. A combination of these factors, as well as the appropriate mode of delivery (intravenous administration or oral administration), could be required to maximize the effects, which is a possible reason why HDIVC treatment in the ICU is sometimes ineffective or has effects opposite to our expectations [18].

## 8. Updating Pauling’s Vitamin C Therapy

### 8.1. Long-Lasting Debates on the Pharmacological Effects of Vitamin C

Ascorbate was proven by Szent-Györgyi to function as vitamin C against scurvy [186], but its function against infectious diseases had been suggested as early as this discovery [187,188,189]. Nonetheless, vitamin C may be more strongly linked to the name of Linus Pauling. In 1971, Pauling proposed that the oral intake of vitamin C may reduce the incidence and morbidity of the common cold based on his own meta-analysis (which is one of the earliest ones), using clinical trial data available at that time [190,191]. Almost simultaneously, Pauling advocated in 1970 the efficacy of vitamin C on the common cold to the public in his book, *Vitamin C and the Common Cold* [192]. Since the clinical trial data that Pauling used were limited, unavoidably biased results were included in these publications, one of the many reasons for the active scientific and political debates on the efficacy of vitamin C on the common cold and other diseases since the 1970s.

Even half a century later, active debates on this issue are still ongoing [193], a seeming “never ending story”. Vitamin C therapy is rejected by many medical professionals today. In discussing why medical professionals are not enthusiastic about the observation that vitamin C decreases the incidence of the common cold by 31%, Pauling wrote, “In the search for a drug to combat a disease the effort is usually made to find one that is 100 percent effective” [194]. He continued, “Also, there seems to have existed a feeling that the intake of vitamin C should be kept as small as possible, even though this vitamin is known to have extremely low toxicity” [194]. Pharmaceutical drugs, including antiviral drugs, are expected to have a high efficacy at low concentrations, because drugs are usually highly toxic at higher concentrations. High-dose administration of drugs is often dangerous and even fatal due to potential side effects. Even at relatively low concentrations, serious side effects do not seem to be uncommon. For example, drug-induced liver damage is problematic in the treatment of COVID-19 [195,196,197]. There is also the case for vaccines [198]. In contrast, vitamin C has virtually no toxicity, even at saturating concentrations in the body [194].

### 8.2. High Dose Necessary for Pleotropic Function of Vitamin C

Pauling emphasized the importance of maintaining high vitamin C concentrations in the body to maintain general health, for example, to prevent the common cold [194]. However, the recommended daily allowance (RDA) issued by the FDA was much less than the amounts that Pauling and his forerunners proposed. In his book, Pauling explained that the RDA value (1980 edition from the FDA) was 60 mg for adult males, but the recommendations of his forerunners and himself were more than 1000 mg [194]. The large difference between these recommended amounts stems from the fact that the RDA value issued by the FDA was set just to prevent scurvy. Szent-Györgyi and Pauling shared the opinion that the optimum intake of vitamin C is much larger than the RDA. Responding to an inquiry from Pauling, Szent-Gyorgyi wrote: “Scurvy is not the first sign of the deficiency but a premortal syndrome” [194]. Unfortunately, the misconception that vitamin C is only for scurvy has prevailed, which appears to have hampered awareness of the pleotropic physiological functions of vitamin C. Now, numerous lines of evidence have accumulated for the pleotropic functions of vitamin C other than prevention of scurvy [199,200,201,202]. Reflecting the diverse functions of vitamin C, together with individual variation, the high levels of vitamin C doses recommended by Pauling (1986) (1000–18,000 mg) [194] cannot be considered unreasonable. The precise values of the recommended daily intake of vitamin C for a given individual should vary based on genetic background, quality of diet, and severity of illness. 

### 8.3. Updating Pauling’s Concept

Pauling discovered the molecular mechanisms of sickle cell anemia and coined the term “molecular disease” [203,204]. The seminal paper, “Sickle cell anemia, a molecular disease” [203], was published in *Science* in 1949, which opened an entirely new field of molecular biology and medicine. However, Pauling did not pay much attention to “molecular medicine”, probably because he had noticed how difficult it is to achieve effective molecular treatments based on molecular mechanisms.

According to the current paradigm in pharmacology, pathogenic microorganisms should be targeted by synthetic drugs that interact with the specific molecules of pathogens to kill them. This paradigm is based on Koch’s postulates [205], which were established more than a century ago. This Koch’s postulates-based paradigm may further make vitamin C less acceptable for medical professionals because Pauling’s vitamin C therapy is based on a paradigm entirely different from the current theories.

In “Western or modern” medicine, a disease can be defined as dysfunction of a physiological mechanism. Based on this concept, a drug in general is presumed to act on a specific component of a physiological mechanism. This way of treating a disease is molecular medicine. In many cases, these are inhibitors of enzymes or transporters, showing the “one-to-one” relationship between the drug and target molecule, in accordance with a structure-function relationship. The “one-to-one” philosophy in medicine and pharmacology works well if the cause of a disease is ascribed to a single component, such as a protein or an enzyme. However, most diseases that are difficult to prevent and cure are syndromes that are governed by multiple components with complicated interactions. COVID-19 is a good example.

Looking back on a long research history, one can notice that our society’s recognition of the simple molecules (nitrite, nitrate, NO and O_2_) has changed over time: harmful or beneficial? There have always been contradictory findings and interpretations regarding the physiological functions of those ubiquitous molecules. Vitamin C also exhibits harmful effects through the chemical Fenton reaction that produces highly toxic ROS (•OH, hydroxyl radicals) [178]. The excessive intake of DHA (the oxidized form of ascorbate) into the cells may cause oxidative stress conditions where the reductants GSH and NADPH are used up for ascorbate regeneration [206], which might be avoided by the supplementation of the glutathione precursor cysteine [207]. Two opposing effects of those simple molecules may lead non-specialists to a state of confusion: is it good for us or bad for us? In addition, the circularity of the relationship, such as between nitrite and NO (Figure 2 and Figure 3), gives rise to the chicken and egg issue: substate or product?

In biochemistry and molecular biology, major target molecules are considered relatively stable, large, and unique in structure, such as proteins, sugars, lipids, DNA, or RNA. These molecules may serve as excellent targets of molecular medicine. The nature of NO and nitrite are opposed to these conventional biomolecules: unstable, small, simple, and ubiquitous. Owing to these properties, conventional approaches are generally not applicable to understanding the physiological functions of these ubiquitous molecules. According to Pauling [131,194], “optimal molecular concentrations of biological substances that are normally present in the body, such as vitamin C, are critical for the function of organs”. Pauling further argued that the optimal concentrations may vary from individual to individual [131,194]. 

For understanding the physiological or pharmacological functions of those simple and universal biomolecules, knowing their fine balance is more valuable than the strict categorization of their effects or targets. Fritjof Capra, a physicist, introduced the parallelism between modern physics and Eastern philosophes in his book *The Tao of Physics* [208]. Chinese medicine is based on traditional Chinese philosophies that are mutually exclusive to “Western” science [209]. Pauling appears to share a similar philosophy with such Eastern philosophies: knowing the fine balance would be the ultimate answer.

Recent studies have revealed that H_2_S/HS^-^ is also involved in the regulation of physiological processes through cysteine thiol persulfidation (Cys-SSH) [210] and polysulfidation (Cys-RSS_(n)_H) [211]. Such reactive sulfur molecules are termed reactive sulfur species (RSS) [57,91]. Complicated chemical interactions between ROS, RNS, and RSS are pronounced in cysteine thiols [57,210]. It is interesting to note that, in addition to nitrite and ascorbate, the sulfur-containing compound thiocyanate (SCN^-^) is present in saliva and gastric juice [212,213,214]. The H_2_S chemical donor isothiocyanates are rich in cruciferous vegetables (Cruciferae or Brassicaceae), such as cabbage, and they have been reported to reduce many types of diseases [215]. It is obvious that RSS also plays an important role in the optimization of the redox balance [91,127]. Updating Pauling’s concept, we propose that the optimal concentrations of ROS, RNS, and RSS that are normally present in the body, such as H_2_O_2_, NO and H_2_S, are critical for the function of organs, especially in vascular systems and immune systems. 

## 9. Conclusions

Continuous exposure to a high concentration of NO may be harmful to host cells, as seen during inflammation. In contrast, a short burst of NO at high concentrations (hundreds ppm) should have maximal antiviral effects on COVID-19 without damaging the host cells, as recently discussed by Del Sorbo et al. (2022) [216]. We conclude that an intermittent NO burst, chemically generated by the combination of nitrite (or nitrate) and vitamin C (or vitamin P, phytophenolics and betalains), is a potential therapeutic treatment to prevent and mitigate COVID-19. Thus, the reductive NO-generating mechanism is worthy of consideration for developing clinical treatments and for designing and interpreting data from clinical trials [217]. It is important to note that chronic diseases associated with the dysfunction of vascular systems, such as hypertension [218], are also expected to be improved by the application of the reductive NO generation strategy. This can be achieved by the daily consumption of fresh green leafy vegetables (nitrate) along with fruits (rich in vitamins C and P and organic acids).

In the application of the reductive NO-generating strategy for clinal treatments, knowing an optimal dose balance of the key molecules (ascorbate, nitrate, nitrite, cysteine) for individuals will be critical to gain the maximum therapeutic effects. The gut microbiota has been interpreted in terms of a metabolic organ that influences the host through reciprocal interactions, encompassing the NO-nitrite-nitrate metabolic and immune pathways in a diet-dependent manner that shapes all aspects of host physiology [219]. Hence, redox metabolism can be strongly influenced by the bacterial communities colonized in the oral cavity and the gut, a situation analogous to the dynamics of bacterial flora in soils [220]. Unfortunately, we have not yet found measurable parameters to monitor the redox balance in cells, tissues, organs, and the whole body. A recent pilot study reported that dogs can distinguish the respiratory secretions of patients with COVID-19 from those of healthy controls, with high rates of sensitivity and specificity [221], implying that there are differences in the volatile compounds between them. To monitor an internal redox status of individuals in a noninvasive manner, real-time analysis of exhaled gases and/or volatile organic compounds (VOCs) will be a hopeful candidate in personalized medicine [222].

## Data Availability

Not applicable.
